# Association between serum non-high-density lipoprotein cholesterol and cognitive dysfunction after acute ischemic stroke: a cross-sectional study

**DOI:** 10.1590/1414-431X20209487

**Published:** 2020-10-30

**Authors:** Yinghui Jiao, Tian Tian, Shasha Wei, Chengdong Wang, Lili Wu

**Affiliations:** 1 Department of Neurology, Weifang People’s Hospital, Weifang, Shandong, China; 2 Operating Room, Weifang Brain Hospital, Weifang, Shandong, China; 3 Prenatal Diagnosis Laboratory, Weifang People’s Hospital, Weifang, Shandong, China

**Keywords:** Non-high-density lipoprotein cholesterol, Ischemic stroke, Cognitive dysfunction, Mini-Mental State Examination scale, Montreal Cognitive Assessment scale

## Abstract

This study aimed to explore the association between serum non-high-density lipoprotein cholesterol (non-HDL-C) and cognitive dysfunction risk in patients with acute ischemic stroke (AIS). This cross-sectional study enrolled 583 AIS patients. Biochemical markers and lipid profile were collected after admission. AIS patients were classified into high group (non-HDL-C ≥3.4 mM) and normal group (non-HDL-C <3.4 mM). Mini-Mental State Examination scale (MMSE), Montreal Cognitive Assessment scale (MoCA), Activities of Daily Living (ADL) scale, Neuropsychiatric Inventory (NPI), and Hamilton Depression scale 21 version (HAMD-21) were applied on the third day after admission. Compared with the control group, patients of the high group had higher body mass index and higher frequency of intracranial artery stenosis, and exhibited higher levels of non-HDL-C, total cholesterol, triglycerides, low-density lipoprotein cholesterol, homocysteine, fasting blood glucose, and glycosylated hemoglobin (HbA1c), and lower levels of high-density lipoprotein cholesterol (all P<0.05). Compared with the control group, patients of the high group had significantly lower MMSE and MoCA scores (MMSE: 26.01±4.17 *vs* 23.12±4.73, P<0.001; MoCA: 22.28±5.28 *vs* 20.25±5.87, P<0.001) and higher scores on the NPI and HAMD-21 (both P<0.001). MMSE (r=-0.306, P<0.001) and MoCA scores (r=-0.251, P<0.001) were negatively associated with non-HDL-C level. Multivariate regression analysis revealed that non-HDL-C level (OR=1.361, 95%CI: 1.059-1.729, P=0.016) was independently associated with the presence of cognitive dysfunction after adjusting for confounding factors. High serum non-HDL-C level might significantly increase the risk of cognitive dysfunction after AIS.

## Introduction

Vascular cognitive impairment is one of the most common complications in patients with acute ischemic stroke (AIS) (1). Dyslipidemia has been considered to be a vital risk factor for cardiovascular disease (CVD), and its roles in the occurrence and development of ischemic stroke are still unknown. Dyslipidemia may not only result in the insidious, progressive decline of vital organ function, but also influence the cognitive function of AIS patients through accelerating systemic atherosclerosis. It has been regarded as a risk factor for inducing cognitive impairment and vascular dementia (2).

Previous consensus and guidelines have recommended low-density lipoprotein cholesterol (LDL-C) as the primary target of lipid-lowering therapy for primary prevention of CVD (3). However, some recent studies show that serum non-high-density lipoprotein cholesterol (non-HDL-C) is more strongly related to cardiovascular or cerebrovascular events than LDL-C level. In 2015, the National Lipid Association recommended that non-HDL-C was more suitable as the primary therapeutic target than LDL-C in the patient-centered management of dyslipidemia. Serum non-HDL-C is defined as the difference between total cholesterol (TC) and high-density lipoprotein cholesterol (HDL-C), including LDL-C, intermediate density lipoprotein-cholesterol (IDL-C), very low density lipoprotein-cholesterol (VLDL-C), chylomicron remnants, and lipoprotein (a). Non-HDL-C contains all of the potentially atherogenic lipid particles, therefore, it is more likely to reflect the risk of atherosclerotic disease (4).

Nevertheless, there is still a lack of studies regarding the roles of dyslipidemia during the acute phase of stroke. Therefore, the present study was conducted to explore the association between serum non-HDL-C level and the risk of developing cognitive dysfunction in a Chinese population of AIS patients.

## Material and Methods

### Patients and study design

This is a retrospective cross-sectional study of hospitalized patients treated for AIS between January 2013 and December 2018 at the Department of Neurology, Weifang People’s Hospital (China). The inclusion criteria were: 1) patients were in accordance with the diagnostic criteria of ischemic stroke according to the Chinese guideline for prevention and treatment of cerebrovascular disease, and computed tomography (CT) or magnetic resonance imaging (MRI) scanning confirmed the new cerebral infarction; 2) time from onset of AIS ≤7 days; and 3) >18 years of age. The exclusion criteria were: 1) patients with disturbance of consciousness, hemiplegia or severe aphasia, and could not complete neuropsychological testing; 2) patients with depression, Lewy body dementia, Alzheimer’s disease, frontotemporal dementia, or dementia induced by other reasons such as intracranial infections, malignant tumors, traumatic brain injury, neurodegenerative diseases, etc.; 3) patients with other severe organ dysfunction; 4) patients with a history of mental illness or abnormal behavior; 5) patients who had ischemic stroke history combined with cognitive dysfunction before the onset of the present AIS; and 6) usage of nootropics or antipsychotics within 4 weeks.

All patients or their legal proxies provided written informed consent before evaluation of neuropsychiatric scales. The study was approved by the Ethics committee of Weifang People’s Hospital (China). Written informed consent to participate in this retrospective study was not considered necessary by the Ethics Committee.

### Clinical data collection

Clinical data of all patients were recorded at admission, including age, gender, education years, family history of dementia, history of stroke, smoking history, and comorbidities such as hypertension, diabetes, and coronary heart disease (CHD). Body mass index (BMI) was calculated as body weight in kilograms divided by the squared height in meters (kg/m^2^). Hypertension was defined as systolic blood pressure ≥140 mmHg and/or diastolic blood pressure ≥90 mmHg, or currently taking antihypertensive medication prescribed by a physician. Patients were defined as having diabetes mellitus if they had been receiving insulin or oral hypoglycemic drugs, if fasting blood glucose (FBG) levels were ≥126 mg/dL, or if patients had been informed of the diagnosis prior to this study. For smoking history, current smokers and patients who had quit smoking within 5 years were considered as positive. For drinking history, patients who had consumed 50 mL or more of alcohol per day for more than 1 year were considered as positive. The clinical severity of the AIS was evaluated by the National Institutes of Health Stroke scale (NIHSS) on admission. Intracranial artery stenosis (ICAS) was defined as the presence of signs of stenosis of the internal carotid artery, middle cerebral artery, anterior cerebral artery, posterior cerebral artery, basal artery, and/or intracranial segment of the vertebral artery. The atherosclerotic stenosis was defined as >50% stenosis of the above main intracranial arteries (5). According to the classification criteria of Oxfordshire Community Stroke Project (OCSP) (6), AIS patients were categorized into three subtypes: total/partial anterior circulation infarction, posterior circulation infarction, and lacunar cerebral infarction.

### Laboratory measurements

Blood samples were collected after overnight fast on the next morning after hospital admission. ADVIA-2400 full automatic biochemical analyzer (Siemens, Germany) and triglyceride test kit (GPOPAP, Shanghai Huachen, China) were applied to measure FBG, total cholesterol (TC), triglycerides (TG), high-density lipoprotein cholesterol (HDL-C), and LDL-C levels. BNP II protein analyzer (Siemens) was used for detection of serum hypersensitive C-reactive protein (hs-CRP), glycosylated hemoglobin (HbA1c), and homocysteine (Hcy) levels.

### Grouping

Non-HDL-C was calculated as TC minus HDL-C. We classified the patients into two groups on the basis of the criteria recommended by patient-centered management of dyslipidemia, which was developed by the National Lipid Association in 2015 (4): high group (non-HDL-C ≥3.4 mM) and normal group (non-HDL-C <3.4 mM).

### Evaluation of neurological function

All patients were treated with conventional treatments such as antiplatelet agents, anticoagulation, and dilatation after admission (7). On the third day of hospital admission, cognitive functions were measured using Montreal Cognitive Assessment scale (MoCA) (8) and Mini-Mental State Examination scale (MMSE) (9). The total score of MMSE was 30 points, of which <17 points in illiterate patients, <20 points in patients with primary school education, and <24 points in patients who attended middle school or higher were regarded as cognitive dysfunction. The total score of MoCA was 30 points. Patients with MoCA below the cutoff score of 26 points were considered as having cognitive dysfunction. In patients with an education level ≤12 years, one point was added to the total MoCA score to correct for educational bias. The Neuropsychiatric Inventory (NPI) was used to measure the frequency and severity of behavioral disturbances in patients with AIS (10). The Activities of Daily Living (ADL) scale was applied to evaluate the functional abilities of patients (11). The Hamilton depression scale 21 version (HAMD-21) is a standard instrument used for determining emotional state (12). For MMSE and MoCA, higher scores indicated better cognitive function. For NPI, ADL, HAMD-21, higher scores indicated greater impairment. The neuropsychological scales were routinely evaluated by trained and experienced neurologists who were blinded to the clinical data and laboratory results.

### Statistical analysis

SPSS 17.0 (IBM, USA) was used for all analyses. The distribution of continuous data was assessed using the Kolmogorov-Smirnov test. Normally-distributed continuous data are reported as means±SD and compared by independent-samples *t*-test between the two groups. Non-normally distributed data are reported as median (interquartile range, IQR) and analyzed using the Mann-Whitney U test. Categorical variables are reported as frequencies and were analyzed using chi-squared test or Fisher’s exact test. The correlations between non-HDL-C level and other clinical characteristics were evaluated by Pearson correlation. Receiver operating characteristic (ROC) curve and the area under the ROC curve (AUC) were also performed to evaluate sensitivity and specificity of non-HDL-C for identification of cognitive dysfunction. Logistic regression (enter method) was used to analyze potential risk factors associated with cognitive dysfunction in patients with AIS. Two-sided P-values <0.05 were considered to be statistically significant.

## Results

### Clinical characteristics of the patients

According to the inclusion and exclusion criteria, 583 AIS patients who were hospitalized at the Department of Neurology, Weifang People’s Hospital between January 2013 and December 2018 were included in the final analysis. Based on non-HDL-C levels, patients were divided into two groups. The normal group had 204 patients (148 men and 56 women), and the mean age was 61.2±7.35 years. The high group included 379 cases (273 men and 106 women), and the mean age was 62.4±8.27 years. There were no significant differences in gender, age, NIHSS at admission, education level, comorbidities, smoking status, drinking history, family dementia history, and previous stroke history between normal group and high group patients (all P>0.05). However, BMI of patients in the normal group was significantly lower than that in high group (P<0.001). Patients of the high group showed a higher ratio of ICAS (P=0.007). In addition, there was no significant difference in the distribution of OCSP subtypes between the two groups (all P>0.05). The results are reported in Table 1.

**Table 1:** Characteristics of the patients of the normal and high non-high-density lipoprotein cholesterol groups.

Variables	Normal group (n=204)	High group (n=379)	P value
Male, n (%)	148 (72.5%)	273 (72.0%)	0.894
Age (years)	61.2±7.35	62.4±8.27	0.073
BMI (kg/m^2^)	24.5±2.5	25.4±3.1	<0.001
NIHSS at admission, median (IQR)	4 (2-9)	6 (4-9)	0.231
Education level (years)	9.2±4.7	9.1±3.9	0.795
Hypertension, n (%)	132 (64.7)	258 (68.1)	0.41
Diabetes, n (%)	45 (22.1)	83 (21.9)	0.965
CHD, n (%)	30 (14.7)	68 (17.9)	0.319
History of smoking, n (%)	71 (34.8)	144 (38.0)	0.472
History of drinking, n (%)	49 (24.0)	98 (25.9)	0.626
Family history of dementia, n (%)	8 (3.9)	15 (4.0)	0.983
Previous history of stroke, n (%)	65 (31.9)	126 (33.2)	0.734
OCSP subtypes			0.255
TACI/PACI, n (%)	130 (63.7)	234 (61.7)	
POCI, n (%)	53 (26.0)	88 (23.2)	
LACI, n (%)	21 (10.3)	57 (15.0)	
ICAS	30 (14.7)	92 (24.3)	0.007

BMI: body mass index; NIHSS: National Institutes of Health Stroke scale; OCSP: Oxfordshire Community Stroke Project; IQR: interquartile range; CHD: coronary heart disease; TACI: total anterior circulation infarction; PACI: partial anterior circulation infarction; POCI: posterior circulation infarction; LACI: lacunar infarction; ICAS: intracranial artery stenosis. *t*-test, Mann-Whitney U test, chi-squared test, or Fisher’s exact test were used.

### Comparison of serum biochemical parameters between normal and high groups

Table 2 shows the serum biochemical parameters between the normal group and the high group. Patients of the high group exhibited higher levels of non-HDL-C, TC, TG, LDL-C, Hcy, FBG, and HbA1c, and lower levels of HDL-C than AIS patients of the control group (all P<0.05). No significant difference was observed for hs-CRP level between the 2 groups (P=0.172).

**Table 2:** Comparison of serum biochemical parameters between normal and high non-high-density lipoprotein cholesterol groups.

Variables	Normal group (n=204)	High group (n=379)	P value
Non-HDL-C (mM)	2.75±0.41	4.39±0.73	<0.001
TC (mM)	3.72±0.71	5.49±0.27	<0.001
TG (mM)	1.28±0.59	1.93±0.42	<0.001
HDL-C (mM)	1.13±0.22	1.07±0.31	0.015
LDL-C (mM)	2.17±0.51	3.38±0.63	<0.001
Hcy (mM)	14.31±3.47	15.95±4.21	<0.001
hs-CRP (mg/L)	4.34±0.61	4.50±2.12	0.172
FBG (mM)	6.02±1.76	6.45±2.71	0.021
HbA1c (%)	6.51±1.01	6.80±1.78	0.012

Non-HDL-C: non-high-density lipoprotein cholesterol; TC: total cholesterol; TG: triglyceride; HDL-C: high-density lipoprotein cholesterol; LDL-C: low-density lipoprotein cholesterol; Hcy: homocysteine; hs-CRP: high-sensitivity C-reactive protein; FBG: fasting blood glucose; HbA1c: glycated hemoglobin. *t*-test was used for statistical comparison.

### Comparison of neuropsychological scale scores between normal and high groups

The MMSE and MoCA scores of patients in the high group were significantly lower than those in the normal group (MMSE: 26.01±4.17 *vs* 23.12±4.73, P<0.001; MoCA: 22.28±5.28 *vs* 20.25±5.87, P<0.001). Moreover, patients of the high group had higher scores on the NPI and HAMD-21, compared with patients of the normal group (both P<0.001), indicating a greater occurrence of cognitive dysfunction and functional deterioration in AIS patients with high non-HDL-C level. There was no significant difference of the ADL score between the two groups (P=0.089). The results of various neurological functional domains are shown in Table 3.

**Table 3:** Comparison of neuropsychological scale scores of patients between the normal and high non-high-density lipoprotein cholesterol groups.

Variables	Normal group (n=204)	High group (n=379)	P value
MMSE	26.01±4.17	23.12±4.73	<0.001
MoCA	22.28±5.28	20.25±5.87	<0.001
NPI	3.25±1.54	6.23±1.19	<0.001
HADM-21	4.39±1.86	6.08±2.08	<0.001
ADL	25.62±3.34	26.21±4.97	0.089

MMSE: Mini-Mental State Examination; MoCA: Montreal Cognitive Assessment scale; NPI: Neuropsychiatric Inventory; HADM-21: Hamilton depression rating scale 21-item; ADL: Activities of Daily Living scale. *t*-test was used for statistical comparison.

### Relationship between non-HDL-C level and other clinical characteristics

Pearson correlation analyses indicated that BMI (r=0.374, P=0.015), FBG (r=0.126, P=0.027), and HbA1c (r=0.183, P=0.039) were positively correlated with non-HDL-C level in all AIS patients. The results are shown in Table 4. In addition, the MMSE (r=-0.306, P<0.001) and MoCA scores (r=-0.251, P<0.001) were negatively associated with non-HDL-C level in all AIS patients.

**Table 4:** Correlations between non-high-density lipoprotein cholesterol level and BMI, glycemic level, and scores of neuropsychological scales.

Variables	Pearson correlation analysis	
	r value	P value
BMI	0.374	0.015
FBG	0.126	0.027
HbA1c	0.183	0.039
MMSE	−0.306	<0.001
MoCA	−0.215	<0.001
NPI	0.276	0.301
HADM-21	0.039	0.427
ADL	0.046	0.276

BMI: body mass index; FBG: fasting blood glucose; HbA1c: glycated hemoglobin; MMSE: Mini-Mental State Examination; MoCA: Montreal Cognitive Assessment scale; NPI: Neuropsychiatric Inventory; HADM-21: Hamilton depression rating scale 21-item; ADL: Activities of Daily Living scale.

### Logistic regression analyses of potential risk factors for cognitive dysfunction after AIS

The potential risk factors for cognitive dysfunction identified by logistical regression in all AIS patients are shown in Table 5. Unadjusted model showed that age, NIHSS at admission, education level, diabetes, family history of dementia, previous history of stroke, ICAS, non-HDL-C level, Hcy level, and HAMD score (All P<0.05) were associated with the risk of cognitive impairment after AIS. All these factors were included in the multivariate regression analysis. Multivariate analysis further revealed that age (OR=1.237, 95%CI: 1.033-1.482, P=0.021), NIHSS at admission (OR=1.013, 95%CI: 1.002-1.024, P=0.023), education level (OR=0.638, 95%CI: 0.440-0.926, P=0.018), previous history of stroke (OR=1.543, 95%CI: 1.031-2.309, P=0.035), ICAS (OR=1.173, 95%CI: 1.014-1.357, P=0.032), non-HDL-C level (OR=1.361, 95%CI: 1.059-1.729, P=0.016), Hcy level (OR=1.203, 95%CI: 1.049-1.379, P=0.008), and HAMD score (OR=1.274, 95%CI: 1.057-1.535, P=0.011) were independently associated with the presence of cognitive dysfunction after AIS.

**Table 5:** Logistic regression analyses of the factors that affected cognitive disorder after acute ischemic stroke.

Variables	Univariate regression analysis			Multivariate regression analysis		
	P	Unadjusted OR	95%CI	P	Adjusted OR	95%CI
Gender						
Female	−	1	Reference			
Male	0.372	0.674	0.283−1.603			
Age (years)	0.002	1.167	1.058−1.287	0.021	1.237	1.033−1.482
BMI (kg/m^2^)	0.718	0.863	0.388-1.920			
NIHSS at admission	0.018	1.125	1.020−1.240	0.023	1.013	1.002−1.024
Education level (years)	0.009	0.275	0.104−0.725	0.018	0.638	0.440−0.926
Hypertension						
No	−	1	Reference			
Yes	0.726	1.372	0.199−2.651			
Diabetes						
No	−	1	Reference	−	1	Reference
Yes	0.008	1.732	1.154−2.599	0.256	1.241	0.855−1.801
CHD						
No	−	1	Reference			
Yes	0.416	1.256	0.725−2.175			
History of smoking						
No	−	1	Reference			
Yes	0.138	1.436	0.890−2.316			
History of drinking						
No	−	1	Reference			
Yes	0.715	1.083	0.706−1.662			
Family history of dementia						
No	−	1	Reference	−	1	Reference
Yes	0.002	1.328	1.109−1.590	0.064	0.429	0.175−1.050
Previous history of stroke						
No	−	1	Reference	−	1	Reference
Yes	0.025	1.267	1.030−1.558	0.035	1.543	1.031−2.309
OCSP type						
TACI/PACI	−	1	Reference			
POCI	0.336	0.714	0.359−1.418			
LACI	0.521	0.682	0.212−2.195			
ICAS	0.025	1.268	1.030−1.561	0.032	1.173	1.014−1.357
TC (mM)	0.321	0.279	0.041−2.512			
TG (mM)	0.745	1.433	0.883−2.325			
LDL-C (mM)	0.064	0.357	0.120−1.062			
HDL-C (mM)	0.574	1.233	0.594−2.559			
Non-HDL-C (mM)	0.027	1.546	1.051−2.275	0.016	1.361	1.059−1.749
Hcy (mM)	0.007	1.152	1.039−1.277	0.008	1.203	1.049−1.379
hs-CRP (mg/L)	0.613	0.872	0.513−1.483			
FBG (mM)	0.347	1.191	0.827−1.714			
HbA1c (%)	0.651	1.249	0.477−3.273			
NPI	0.392	1.421	0.636−3.177			
HADM-21	0.009	1.197	1.046−1.370	0.011	1.274	1.057−1.535
ADL	0.281	1.081	0.938−1.245			

BMI: body mass index; NIHSS: National Institutes of Health Stroke scale; CHD: coronary heart disease; OCSP: Oxfordshire Community Stroke Project; TACI: total anterior circulation infarction; PACI: partial anterior circulation infarction; POCI: posterior circulation infarction; LACI: lacunar infarction; ICAS: intracranial artery stenosis; TC: total cholesterol; TG: triglyceride; LDL-C: low-density lipoprotein cholesterol; HDL-C: high-density lipoprotein cholesterol; non-HDL-C: non-high-density lipoprotein cholesterol; Hcy: homocysteine; hs-CRP: high-sensitivity C-reactive protein; FBG: fasting blood glucose; HbA1c: glycated hemoglobin; NPI: Neuropsychiatric Inventory; HADM-21: Hamilton depression rating scale 21-item; ADL: Activities of Daily Living scale.

### ROC curve for non-HDL-C in identifying cognitive dysfunction in AIS patients

**Figure 1:**
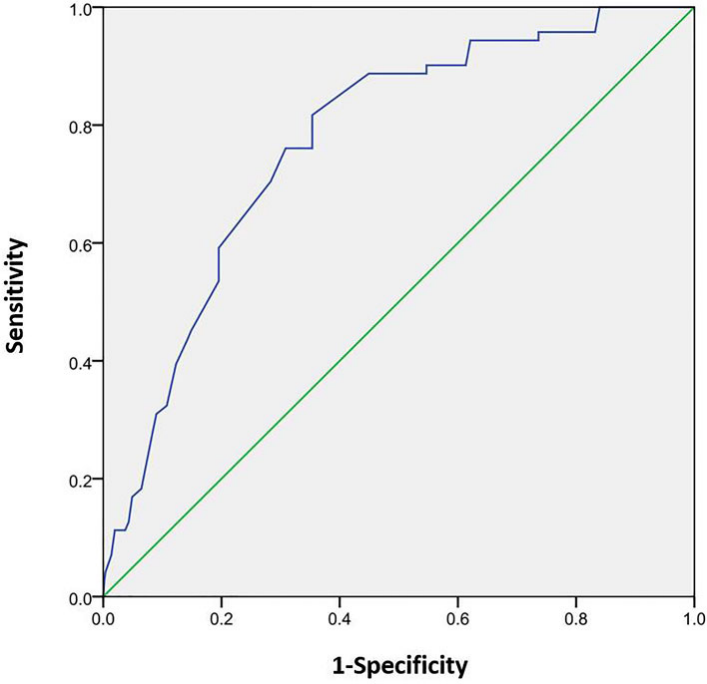
ROC curve was conducted to verify the association of non-high-density lipoprotein cholesterol level with the presence of cognitive dysfunction in acute ischemic stroke patients.

ROC curve was plotted to verify the association between non-HDL-C level and the presence of cognitive dysfunction in AIS patients (Figure 1). The AUC of non-HDL-C for the presence of cognitive dysfunction was 0.773 (95%CI: 0.720-0.826) in AIS patients, and the cut-off value of non-HDL-C level was 3.52 mM. The sensitivity, specificity, positive predictive value, and negative predictive value were 90.3, 63.7, 73.9, and 85.3%, respectively.

## Discussion

Results from some observational cohorts indicate that non-HDL-C is superior to LDL-C for the prediction of both cardiovascular mortality and all-cause mortality (13,14). A clinical longitudinal follow-up study with 2406 men and 2058 women over 19 years indicated that CHD risk in men with non-HDL-C level above 220 mg/dL was 2.14 times that of men with non-HDL-C level lower than 160 mg/dL, and the risk in the subjects with LDL-C levels higher than 190 mg/dL was 1.77 times that of those with LDL-C levels lower than 130 mg/dL (15). The Bypass Angioplasty Revascularization Investigation study tracked the secondary prevention of 1514 patients with multiple coronary lesions. After 5 years of follow-up, non-HDL-C level was still an independent predictor of nonfatal myocardial infarction, while LDL-C had no significant predictive value for endpoint events or mortality (16).

Patients with mild cognitive impairment (MCI) due to ICAS are more likely to develop cognitive deterioration and progression to dementia (17). Possible mechanisms may be that atherosclerosis could not only induce vascular entanglement and distortion, but also result in mechanical obstruction of intracranial macrovascular diseases. Moreover, the presence of ICAS was always accompanied by extensive microvascular disease, systemic atherosclerosis, microcirculatory damage, increased resistance of small vessels, and reduced vascular reactivity, finally resulting in insufficient cerebral perfusion. The additive effects of these mutual hemodynamic dysfunctions might play an important role in accelerating the development and progression of cognitive impairment (17). Previous clinical studies suggested that risk of cerebrovascular disease may be closely related to serum non-HDL-C level. Wu et al. conducted a follow-up study of 95,916 people aged 18-98 years without stroke or myocardial infarction in Tangshan, China. The results showed that serum non-HDL-C level was independently associated with ischemic stroke. High non-HDL-C level could cause more than 50% increase of the ischemic stroke risk in healthy people. In their study, 13.04% of the subjects were diagnosed with asymptomatic ICAS (18,19). Previous studies had demonstrated that elevated serum non-HDL-C level was positively associated with the development of ICAS and an independent risk factor for ICAS occurrence (20,21).

In addition, some studies also indicate that serum non-HDL-C level of MCI patients is statistically higher than that of a population with normal cognitive function, and scores of cognitive function were negatively related with non-HDL-C level. Serum non-HDL-C could be considered to be a reliable predictor of MCI risk in Chinese type 2 diabetic mellitus (T2DM) patients (22). Our study found that 65% of patients with AIS had significantly higher serum non-HDL-C level, and patients of the high non-HDL-C level group showed a higher ratio of ICAS. Non-HDL-C level was positively associated with the occurrence and severity degree of cognitive impairments after AIS. Both serum non-HDL-C level and the presence of ICAS were the independent risk factors for vascular cognitive impairment.

Comparing various neuropsychological scales between the two groups, we found that patients with high serum non-HDL-C level had apparent cognitive impairment, obvious mental behavioral symptoms, and emotional disorder, which could significantly influence quality of life. Multivariate regression analysis demonstrated that higher serum non-HDL-C level was associated with the increased risk of cognitive impairment after AIS. For the identification of cognitive dysfunction in AIS patient, the cut-off value of 3.52 mM for non-HDL-C was suggested in our study. Recent research found that both low HDL-C level and high LDL-C level were associated with deposition of amyloid plaque in the brain, suggesting a close relationship between cholesterol and cognitive dysfunction (23).

The damage of non-HDL-C on cognitive dysfunction after AIS might be associated with its strong atherogenic effects. First, serum non-HDL-C could accurately reflect cholesterol levels of all lipoproteins except HDL-C, because it contains almost all known or potential fat particles with atherosclerotic effects. Each lipid composition has vital predictive value for ischemic stroke and/or vascular stenosis (22,24). Second, the high concentrations of TG and VLDL-C in the composition of non-HDL-C have the potential to produce atherogenic granules in the liver. The interactions of these lipid particles with liver receptors are weakened, and the scavenging ability is reduced. This results in the longer stay of these lipid particles in the circulating blood, which in turn promotes arteriosclerosis. Finally, some of the triglyceride-rich lipoproteins could enter the arterial walls, leading to the development and progression of atherosclerosis (25,26). Therefore, compared with non-HDL-C, using LDL-C alone might ignore the induction of ischemic stroke by other lipid particles.

Our study also indicated that elevated serum non-HDL-C was accompanied with increased serum Hcy and blood glucose levels. Previous studies had indicated that type 2 diabetes mellitus (T2DM) patients often have abnormal lipid metabolism, especially those with poor glycemic control. Abnormal lipid metabolism is mainly related to diabetic angiopathy. Specific lipid-lowering therapy or a low-fat diet could prevent or delay the aggravation of diabetic angiopathy, indicating that T2DM and lipid metabolism dysfunction might share common pathophysiological mechanisms for post-stroke cognitive impairment (27). In addition, diabetic patients often have combined impaired renal function, and studies show that patients with reduced glomerular filtration rate have a higher tendency for cognitive impairment (28). However, the relationships between all these factors are complicated, and further studies are required for comprehensive evaluations.

Multivariate regression analysis indicated that Hcy as a metabolic disorder parameter was also independently associated with the risk of cognitive dysfunction in AIS patients, which might be related to homocysteine-mediated neurotoxicity (29). In addition, our results demonstrated that low education level, advanced age, NIHSS at admission, high HAMD score, previous stroke history, and family dementia history were independently associated with cognitive dysfunction after AIS, which were in line with previous studies (24,30).

The present study had several limitations. First, this was a single-center study with a small sample size, so the generalizability of the results remains unknown and the study might be underpowered to detect real differences between groups. Second, this was a cross-sectional study, and the cause-effect relationship between non-HDL-C and cognitive impairment could not be drawn. Third, scores of individual cognitive tests were not transformed to z-scores (i.e., normalized scores) for further analysis in our AIS patients, which might have been prone to selection bias or information bias. However, the z-score models of cognitive impairment screening scales were unavailable in the Chinese population. Fourth, we only detected cognitive performance on the third day after hospital admission. Future studies should explore the fluctuations of cognitive impairment during the sub-acute phase and chronic phase in a larger cohort of AIS patients over a longer follow-up period.

In conclusion, our cross-sectional study suggested that high serum non-HDL-C level might significantly increase the risk of cognitive dysfunction after AIS. As an easy-to-test and inexpensive biomarker, non-HDL-C screening could be conducted for primary prevention of cognitive impairment after AIS in the Chinese population.
